# The Challenges of Detecting Circulating Tumor Cells in Sarcoma

**DOI:** 10.3389/fonc.2016.00202

**Published:** 2016-09-07

**Authors:** Marta Tellez-Gabriel, Hannah K. Brown, Robin Young, Marie-Françoise Heymann, Dominique Heymann

**Affiliations:** ^1^UMR 957, Pathophysiology of Bone Resorption and Therapy of Primary Bone Tumours, Equipe Ligue 2012, Faculty of Medicine, INSERM, University of Nantes, Nantes, France; ^2^Laboratotio Hematologia Oncologica y de Transplantes, Institut Investigacions Biomèdiques (IBB) Sant Pau, Hospital de la Santa Creu i Sant Pau, Barcelona, Spain; ^3^Department of Oncology and Metabolism, Medical School, University of Sheffield, Sheffield, UK; ^4^European Associated Laboratory, INSERM-University of Sheffield, Sarcoma Research Unit, Medical School, Sheffield, UK; ^5^Nantes University Hospital, Nantes, France

**Keywords:** sarcoma, neuroblastoma, rare cancers, circulating tumor cells, cancer stem cells

## Abstract

Sarcomas are a heterogeneous group of malignant neoplasms of mesenchymal origin, many of which have a propensity to develop distant metastases. Cancer cells that have escaped from the primary tumor are able to invade into surrounding tissues, to intravasate into the bloodstream to become circulating tumor cells (CTCs), and are responsible for the generation of distant metastases. Due to the rarity of these tumors and the absence of specific markers expressed by sarcoma tumor cells, the characterization of sarcoma CTCs has to date been relatively limited. Current techniques for isolating sarcoma CTCs are based on size criteria, the identification of circulating cells that express either common mesenchymal markers, sarcoma-specific markers, such as CD99, CD81, or PAX3, and chromosomal translocations found in certain sarcoma subtypes, such as EWS-FLI1 in Ewing’s sarcoma, detection of osteoblast-related genes, or measurement of the activity of specific metabolic enzymes. Further studies are needed to improve the isolation and characterization of sarcoma CTCs, to demonstrate their clinical significance as predictive and/or prognostic biomarkers, and to utilize CTCs as a tool for investigating the metastatic process in sarcoma and to identify novel therapeutic targets. The present review provides a short overview of the most recent literature on CTCs in sarcoma.

## Introduction

Sarcomas are a heterogeneous group of soft tissue and bone neoplasms that arise from mesoderm or ectoderm (1) and consequently may arise from mesenchymal stem cells (2). Helman and Meltzer (3) associated different molecular alterations with specific histological entities and suggested that sarcomas can be defined by their molecular signatures. This observation is strengthened by recent publications identifying a specific subgroup of thoracic sarcomas based on SMARC4A inactivation (4) and a “BRCA-ness” signature in osteosarcomas (5). These molecular signatures include sarcoma-specific translocations that result in oncogenic fusion genes, which are believed to be necessary for malignant transformation and are utilized for molecular-based subgrouping.

**Figure 1:**
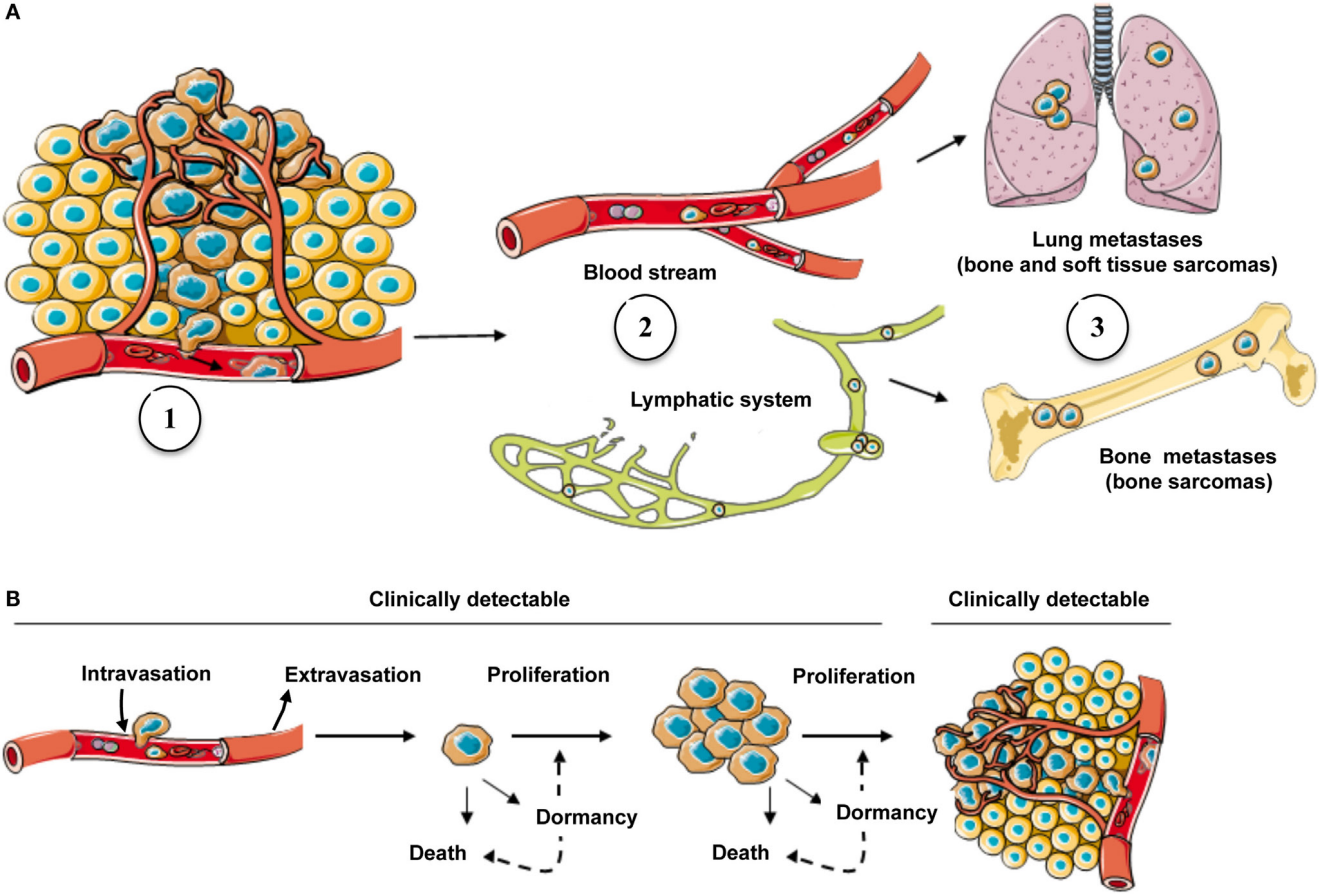
**The metastatic process in sarcoma and possible fates of cancer cells in secondary site**. **(A)** Cells escaping from the primary tumor into the blood circulation (1) are carried by the flow, either blood stream or lymphatic system (2), to secondary sites where they grow if they find a favorable environment (3). **(B)** Following the arrival of CTCs into a secondary organ, only a subset will survive and generate metastases (clinically detectable) and remainder cells might either go into a state of dormancy/quiescence or die (clinically undetectable).

Distant metastases develop in half of sarcoma patients presenting initially with localized disease, with the lungs being the most common metastatic site (1). The vast majority of sarcomas, excepted epithelioid sarcoma, angiosarcoma, and alveolar rhabdomyosarcoma, which can invade regional lymph nodes, predominantly spread through the blood vasculature. This modality is not exclusive and can be associated with spread from the lymphatic system into the blood vasculature (6). To generate metastases, tumor cells must overcome several constraints: escape from the primary site through the invasion of cancer cells from the basal membrane into a blood or lymphatic vessel, a process called intravasation (7); survival in the circulation; arrest in the capillaries at a new site; migration from the capillary into the interstitial space; and establishment of tumor growth at the new location (Figure 1). As these steps are sequential and dependent on each other, only a small number of cells will successfully complete all of them, illustrating the considerable inefficiency of the metastatic process (8). Interestingly, communications between tumor cells and the host tissue play an important role in the establishment and development of metastatic foci (9–11).

Current methods to detect tumor recurrence or the development of metastasis is largely dependent on clinical examination and/or radiographic imaging to identify the location/expansion of tumor growth, such as computerized tomography (CT), which uses multiple X-rays to produce cross-sectional layers that show detailed images inside the body, including bones, organs, tissues, and tumors (12), or positron emission tomography based on the injection, inhalation, or swallowing of radioactive tracers. Metabolic disturbances associated with tumor growth can be then detected by these techniques (13). These approaches present some advantages: (i) they are painless, easy to set up, and rapid; (ii) they can help in the diagnosis and serve as a guide for treatment; (iii) they can be used in the treatment follow-up. Nevertheless, these methodologies show also important disadvantages such as (i) exposure to ionizing radiation (X-ray or gamma rays) with potential risks of secondary cancer; (ii) injection of a contrast medium (dye) can cause kidney problems or result in allergic/injection-site reactions in some patients; and (iii) some procedures require anesthesia (14–16). New methods are needed to enable the earlier detection of tumor recurrence and metastasis, and to improve the diagnosis, treatment, and surveillance of patients suffering from sarcoma. Detection of circulating tumor cells (CTCs), as a measure of metastatic potential, could provide a way to target a patient population more likely to benefit from adjuvant chemotherapy. To date, however, the clinical significance of CTCs, as a prognostic or predictive marker in sarcoma, is uncertain.

Circulating tumor cells can be detected in the peripheral blood and in theory have the potential to extravasate to form tumor metastases (17). CTCs are cells that circulate in the peripheral blood, while disseminated tumor cells (DTCs) are cells located in secondary organs such as bone marrow. DTCs then derive from CTCs. Interestingly, Kim et al. (18) suggested that DTCs converted into CTCs can also return to and enrich the primary tumor, a process termed “tumor self-seeding” or “cross-seeding.” This process was confirmed recently by Gundem et al. (19). Detection of CTCs has been then investigated across numerous tumor types but most commonly in epithelial neoplasms (20). The relatively non-invasive nature of CTC isolation and their likely correlation with the metastatic process and potential as a biomarker of disease progression and therapeutic response warrants further investigation as a clinical tool. The present review gives a brief overview of the most recent techniques available for CTC isolation from sarcoma patients.

## Methods for Isolation of CTCs in Patients Suffering from Rare Cancers

Studies of the detection of CTCs in sarcomas are relatively recent due to the limited number of patients, their high diversity/heterogeneity, and the absence of specific markers expressed by sarcoma tumor cells (Table 1).

**Table 1:** **Summary of published studies on circulating tumor cells from sarcoma and neuroblastoma**.

Sarcoma type	Detection method	Method for CTC enrichment/isolation	Marker	Main conclusion	Reference
Ewing’s sarcoma	RT-PCR	Whole blood	EWS-FLI-1/ERG	Detection of CTCs in BM and PB in patients with localized disease. Association with poor outcome	West et al. (21); Schleiermacher et al. (22)
Ewing’s sarcoma	RT-PCR	Whole blood	EWS-FLI-1/ERG	Detection of CTCs in BM and PB correlates with disease progression	Avigad et al. (23)
Ewing’s sarcoma	RT-PCR	Density gradient	EWS-FLI-1/ERG	Detection of tumor cell in BM is associated with reduced survival	Fagnou et al. (24)
Ewing’s sarcoma	RT-PCR	Density gradient	EWS-FLI-1/ERG	No prognostic data	Peter et al. (25); Zoubek et al. (26)
Alveolar soft part sarcoma (ASPS)	RT-PCR	Red blood cells lysis buffer	ASPSCR1–TFE3	Detection of CTCs in PB of patients but not in healthy individuals. Clinical significance must be validated	Hoshino et al. (27)
Rhabdomyosarcoma (ARMS)	RT-PCR	Density gradient	PAX3–FKHR PAX7–KFHR	Detection of minimal disease in PB and BM. Larger number of samples must be analyzed to correlate MRD with clinical relapse	Kelly et al. (28)
Osteosarcoma	RT-PCR	Density gradient	mRNA of osteoblast-related genes	From analysis of peripheral blood, collagen type I had a higher expression in OS patients than in healthy people. Moreover, expression correlated with the development of metastases	Wong et al. (29)
Osteosarcoma	PCR-ELISA	Density gradient	Osf2 mRNA	*Osf2* mRNA was significantly higher in the blood of mice with metastasis than in controls	Hatano et al. (30)
Neuroblastoma	RT-PCR	Density gradient	Tyrosine hydroxylase	Association of CTC expressing high levels of tyrosin hydroxylase with poor prognosis	Burchill et al. (31); Träger et al. (32); Kuroda et al. (33)
Multiple sarcomas (OS, leiomyosarcoma, angiosarcoma, and pleomorphic sarcoma)	Flow cytometry	CD45-positive cellsdepletion	Cell-surface vimentin	CSV as a universal sarcoma CTC marker by using a monoclonal antibody. This marker has not yet been clinically validated	Satelli et al. (34)
Multiple sarcomas	ISET		Cell size	ISET was able to identify CTCs in patients with high-grade sarcoma	Chinen et al. (35)
Ewing’s sarcoma	Flow cytometry	Density gradient	CD99	Bi-color flow cytometry for CD99^+^CD45^−^ cells provides a new strategy for detecting circulating Ewing’s sarcoma cells. The clinical evaluation and validation of this method is ongoing	Dubois et al. (36)
Neuroblastoma	Flow cytometry	Whole blood	CD81 and CD56	Triple-color flow cytometry analysis using CD81/CD56/CD45 is useful for detecting neuroblastoma cell lines in peripheral blood. Further clinical validation of this approach is needed	Nagai et al. (37); Bozzi et al. (38)
			NB84	NB84 marker improves the detection of infiltrating neuroblastoma cells, especially in cases of dubious positivity of CD56 marker	
Rhabdomyosarcoma	Flow cytometry	Red blood cells lysis buffer	PAX3		Almazán-Moga et al. (39)
Soft-tissue sarcoma	Immunocytochemistry	CellSearch System	EPCAM and cytokeratins 9/18/19	Detection of CRT in soft-tissue sarcomas expressing the EpCAM epithelial marker. No demonstration of clinical significance	Vincenzi et al. (40)

*BM, bone marrow; PB, peripheral blood; MRD, minimal residual disease; ES, Ewing’s sarcoma; OS, osteosarcoma; CSV, cell-surface vimentin; CTC, circulating tumor cells; ISET, isolation by size; density gradient, isolation of mononucleated cells*.

### Isolation of CTCs Based on Non-Specific Parameters

Because CTCs are frequently larger than that of normal circulating cells in blood, cell size represents a potential criterion for isolating sarcoma CTCs. Isolation by size of sarcoma cells (ISET, Rarecells Diagnostics, France) was first described by Chinen et al. (35). Authors concluded that size was a “universal” approach for the isolation of CTCs from patients with different types of sarcoma. Filtration methods are relatively rapid, sensitive, and easy technique; nevertheless, the lack of multicentric studies impairs their clinical validity (41, 42). After isolation, CTCs are also characterized by immunocytochemistry and more specifically by the absence of (i) white blood cell markers, such as anti-CD45 (leukocyte common antigen) or anti-CD34 (hematopoietic and vascular-associated tissue marker), and (ii) the absence of epithelial-related markers, such as anti-Pan CK (35).

Another strategy for CTC detection in sarcomas is the use of common mesenchymal cell markers such as vimentin. Satelli et al. developed an anti-vimentin antibody allowing the detection of sarcoma CTCs. This antibody was able to discriminate the expression of cell-surface vimentin, mainly associated with cancer cells, from the intracellular vimentin expressed by white blood cells (34). The authors validated the usefulness of this antibody in different subtypes of sarcoma, such as osteosarcoma, Ewing’s sarcoma, leiomyosarcoma, angiosarcoma, and pleomorphic sarcoma, and defined cell-surface vimentin as a potential universal marker for isolating sarcoma CTCs.

### Use of “Specific” Markers for Isolating CTCs

Circulating tumor cells can more easily be identified in sarcoma subtypes associated with specific chromosomal translocations leading to the expression of a unique fusion product, which is found in tumor cells but not in normal cells. This approach requires an initial pre-enrichment step of CTCs from peripheral blood, which is based more commonly on density gradient. This step allows the recovery of the blood mononuclear fraction where CTCs are present and must include important positive and negative controls to determine the sensitivity of the subsequent assay (28).

The best example of this is the Ewing’s family of tumors, in which the chromosomal translocation (EWS-ERG or EWS-FLI1) can be detected by FISH or the specific fusion gene product can be analyzed by RT-PCR (43). Results from a few small clinical studies of patients with Ewing’s sarcoma (21–24) or neuroblastoma (33) suggest that the detection of CTCs at diagnosis may be associated with worse clinical outcomes and that CTCs may be an early marker of recurrent disease. The fusion gene ASPSCR1–TFE3 can be detected in peripheral blood from patients with metastatic alveolar soft part sarcoma (ASPS) but is undetectable in healthy individuals. The clinical significance of CTCs in ASPS remains to be established (27). Kelly et al. (28) found that the presence of PAX3–FOX1 and PAX7–FOXO1 fusions in CTCs located in bone marrow correlated with clinical outcome in alveolar rhabdomyosarcoma.

Wong et al. (29) described a semi-quantitative RT-PCR for measuring mRNA levels of osteoblast-related genes like in CTCs from peripheral blood of osteosarcoma patients and found that type I collagen levels were significantly higher in osteosarcoma patients than in healthy subjects. Furthermore, high collagen mRNA levels were strongly associated with the subsequent development of clinical metastases and may be a prognostic marker to identify osteosarcoma patients with a high risk of metastasis/recurrence at the time of diagnosis (29). Similarly, Hatano et al. developed a system with a PCR assay based on an enzyme-linked immunosorbent assay (PCR-ELISA) to detect circulating osteosarcoma cells in a mouse metastatic model. This model was characterized by a splicing variant of the transcription factor Osf2 restricted to bone and osteosarcoma (30). The level of this splicing variant was significantly higher in the blood of mice with metastasis than in the control group (30), suggesting that Osf2 mRNA is a potential marker for detecting CTCs in osteosarcoma.

Various tumor cells produce high quantities of specific metabolic enzymes, such as neuroblastomas (sarcoma-related tumors), which produce a large amount of tyrosine hydroxylase, an enzyme that coverts l-tyrosine to l-3,4-dihydroxyphenylalanine (l-DOPA). The expression levels of this enzyme can be measured by RT-PCR in either bone marrow or peripheral blood, as a marker of CTCs, and several studies have associated high levels of tyrosine hydroxylase mRNA with a poor prognosis in neuroblastoma (31, 32).

Multiple studies report the use of specific makers for sarcoma CTC detection by flow cytometry, including CD99 in Ewing’s sarcoma (36); CD81, CD56, and NB84 in neuroblastoma (37, 38); and PAX3 in rhabdomyosarcoma (39). For isolating CTCs, pre-enrichment steps are required in combination with specific antigen recognition for discriminating CTCs from circulating hematopoietic cells (anti-CD45 marker) and epithelial cells (pan-cytokeratin-related marker) (42). In contrast to the previous impression that EpCAM expression was restricted to epithelial tissue and epithelial-derived tumors, a meta-analysis of gene expression profiles demonstrated that EpCAM mRNA was expressed by different sarcoma cell lines (44). Interestingly, subsequent immunohistochemical staining of archived solid tumor samples revealed the expression of EpCAM protein in a subset of angiosarcoma, leiomyosarcoma, and in all the osteosarcoma samples analyzed (44). These works are in agreement with the expression of EpCAM reported on CTCs isolated from sarcoma patients. Vincenzi et al. (40) detected EpCAM-positive CTCs in 43% of metastatic soft-tissue sarcoma patients using the CellSearch System.

## Circulating Tumor Cells vs. Cancer Stem Cells

Cancer stem cells (CSCs) share some similarities with physiological stem cells in terms of self-renewal, production of differentiated progeny, utilization of common signaling pathways, and maintenance of the stem cell niche (8). However, CSCs differ in their tumorigenic activity, as in contrast to physiological stem cells they can induce the formation of tumor masses when transplanted into animals (28, 45).

Several studies suggest that tumor recurrence is due to an increase in CTC number and subsequent transformation of some of these circulating cells into CSCs (46–50). Notable findings in breast cancer demonstrate that a subclone of CTCs express CSC phenotypes (51–53). Moreover, accumulating evidence shows that a subset of CTCs and CSCs exhibit an epithelial–mesenchymal transition (EMT) phenotype (54), enabling these cells to survive in the peripheral blood circulation and actively cause tumor relapse. These findings suggest that EMT links CTCs and CSCs. The hypothesis that a subgroup of CTCs have CSC hallmarks, such as self-renewal and asymmetric cell division, is reinforced by the expression of related molecular markers such as Nanog, Oct4, or Nestin by a subpopulation of CTCs (55, 56). In addition to the commonly known stem cell markers, studies have attempted to identify specific markers that enable the detection of CSCs in sarcoma. To date however, only a few reports have shown the existence of CSCs in bone and soft-tissue sarcoma (57–60). Gibbs et al. found a subset of stem-like cells in bone sarcomas with the capacity to form sarcospheres and to self-renew in culture. Furthermore, they found cells derived from these tumors that express mesenchymal stem cell markers: Stro-1, CD44, and CD105 (57). For the first time, Wu et al. (58) showed the existence of a side population of cells in mesenchymal tumors that was enriched with tumor initiating cells and established a direct correlation between the number of this side population cells and the aggressiveness of the tumors. Another study carried out by Murase et al. (60) demonstrated in several human osteosarcoma cell lines the existence of a side population with self-renewal and cancer-initiating capacity *in vitro* and *in vivo*, supporting the idea that bone sarcomas might contain a population of CSCs. Bian et al. compared the peripheral blood of bone sarcoma patients and healthy subjects and observed a higher quantity of mesenchymal stem cell-like cells in the first group. This increment was accompanied by higher levels of HGF and VEGF in the plasma (59). It has been described that HGF can enhance the proliferation, migration, and invasion potential of osteosarcoma cells (61), and VEGF promotes mesenchymal stem cell proliferation and is involved in angiogenesis and cancer development (62–64).

Greco et al. (65) analyzed a series of bone sarcoma patients and found a correlation between aldehyde dehydrogenase (ALDH) activity and metastatic potential. One study carried out by Martins-Neves et al. suggested the co-existence of different CSC within osteosarcoma, which seemed dependent on the histological subtype. Distinct CSC subsets may assume different functions according to their role in the maintenance of self-renewal (spheres) or chemo-resistance (ALDH activity and side population) (66). Collectively, it appears that CSCs may be present in sarcoma, but the exact composition of these cells and their correlation with CTCs remain to be established.

## Conclusion and Future Perspectives

The CTC domain is technically challenging, as CTCs are very rare with only a few found per milliliter of peripheral blood. They have a highly heterogeneous phenotype and are not the only rare cells in blood; thus for isolation and study, they must be distinguished from epithelial and non-epithelial non-tumor cells, atypical non-tumor cells, endothelial cells, and other rare circulating cells such as stem cells. Many authors have previously isolated CTCs from carcinomas and demonstrated their prognostic value in various carcinomas (17). However, the majority of methods used for isolating these CTCs are based on epithelial antigen-targeted antibodies, and thus they neither allow the isolation of the most malignant CTCs undergoing EMT nor the detection of CTCs from sarcomas (42, 52). In recent years, the technologies for CTC enrichment and molecular characterization have significantly improved. Their application in large clinical trials will not only increase our understanding of the biology of CTCs and their contribution to local recurrence and distant metastasis but will also open another non-invasive route for biomarker discovery. Importantly, the specificity of CTC markers needs to be improved and validated. In sarcomas characterized by chromosomal translocations, where aberrant gene fusion products are constitutively present, this is a relatively straightforward task. As described by Wong et al. in osteosarcoma, the detection of simple overexpression of non-modified genes is much more complex (58). Non-specific markers of osteoblastic differentiation are present in a variety of circulating cells. One way in which Chinen et al. (35) attempted to cut through this background noise was by combining size- and marker-specific approaches (58).

Current (RT)-PCR-based methods to detect tumor cells in peripheral blood and/or bone marrow in soft-tissue sarcomas show several limitations. They are specific to a subtype of sarcoma and cannot be extended to another subtype; their “sensitivity” depends on the level of expression of the targeted transcript in the tumor cells, which can be variable; and their specificity depends on the absence of similar transcripts (in the case of non-mutated transcripts) in peripheral blood cells. Finally, they neither allow a reliable enumeration of CTCs nor to visualize CTC morphology and to study their protein expression and invasive potential.

Vimentin is an interesting marker for the identification of sarcoma CTCs, as it is associated with AKT1 activation in the process of sarcoma tumor cell motility and invasiveness (67). Vimentin expression is not specific to sarcoma CTCs (68). Several authors have reported its expression in carcinoma CTCs, where it is associated with an EMT phenotype, “stemness,” and increased malignant potential (67, 69, 70). Vimentin expression in sarcoma CTCs could be a marker of increased aggressiveness and invasiveness of these tumor cells. Consequently, future studies are required to better understand the biological significance of vimentin expression in CTCs and to correlate its expression with clinical outcomes.

Combining technological strategies for isolating CTCs may enhance the veracity of the results obtained. Gallego et al. (71) analyzed CTCs in patients suffering from either alveolar or embryonic rhabdomyosarcoma, combining the detection of a fusion gene product and muscle-specific markers, including MyoD1 and myogenin. The authors concluded that (i) CTCs were detectable in peripheral blood in a high number of patients, (ii) CTCs detected at the end of treatment were markers of a poorer prognosis, (iii) CTCs preceded the metastatic relapse, and (iv) the detection of CTCs by multiple gene expression was highly efficient and reproducible (71). In a recent study, Satelli et al. (72) compared the detection of CTCs in breast cancer patients by using the CellSearch^®^ (EpCAM-based technique) method and the detection of cell-surface vimentin. They concluded that the summation of CTCs detected by both methods appeared a much stronger and more reliable predictor of therapeutic outcome in metastatic breast cancer patients undergoing therapy. EpCAM may be useful for the isolation of CTCs in sarcoma as part of an approach that combines multiple methodologies (71). Although Cellsearch^®^ is considered as the gold standard for CTC detection methods, new methods of detection based on diverse technical approaches have been recently developed (42, 73). The isolation of CTCs in sarcomas by the detection of membranous makers, such as Cellsearch^®^ and DEPArray™, for instance, is perfectly justified even if the results remain to be validated in clinical practice (42). The key question now is to determine if the isolation of single CTCs is the most pertinent parameter to study compared to the clusters of CTCs, which are potent initiators of metastasis (74, 75).

Circulating tumor cells undoubtedly play an important role in the processes of tumor initiation and progression, as well as during metastasis formation and relapse of the disease (76, 77). Indeed, in heterogeneous tumor tissue, only CSCs are thought to initiate tumor growth after grafting into immunodeficient mice (78). The coexistence of different CSC, as proposed by Martins-Neves et al. (66), implies that therapies targeting CSCs should consider their clonal heterogeneity to enable their effective eradication. Thus, a full characterization of CSCs by tumor subtype may provide key information for the development of new effective anti-neoplastic therapies. Several studies have elucidated the existence of a CSC subpopulation in sarcoma. Findings from studies of various types of sarcoma suggest putative CSCs markers such as Nanog, Oct4, and Nestin (55, 56), as well as Stro-1, CD44, and CD105 (57) in a subpopulation of CTCs. Nevertheless, despite published results especially of various osteosarcoma and rhabdomyosarcoma cell lines, the phenotype of CSCs remains unclear, and their use for diagnostic or therapeutic purposes is ambiguous (79). The identification of a more accurate genetic profiling of this subpopulation of cells could serve to identify new specific markers and therapeutic targets. Further studies will contribute to their characterization in human bone and soft-tissue sarcomas and will help to better understand their pathogenesis (80).

The use of CTCs might be an important diagnostic tool for the earlier detection of metastatic disease for monitoring therapeutic response and for identifying the time point during treatment at which an adjustment in therapy is indicated. CTC analysis of well-annotated patient samples such as those collected during prospective clinical trials will help to develop this exciting field to offer new insights into the pathogenesis of sarcoma and ultimately to improve the future clinical management of sarcoma patients.

## Author Contributions

MT-G, HB, RY, M-FH, and DH contributed equally to the preparation of this review.

## Conflict of Interest Statement

The authors declare that the research was conducted in the absence of any commercial or financial relationships that could be construed as a potential conflict of interest.
